# Have We Not Too Many Dental Schools?

**Published:** 1879-02

**Authors:** 


### HAVE WE NOT TOO MANY DENTAL SCHOOLS?

Would it not be well to reduce to one or two, the dozen dental schools of our country, no one of which is able properly to compensate its teachers for time employed, which they can ill afford from their private practice?

There are evils connected with these, which would be lessened by reducing their number. To attract students to keep them in existence, it seems to be considered necessary to have a low standard of acquirements in students received, and also for graduation. For the same reason the discipline must be lax. Young America must be allowed, if he pleases, to spend more of the lecture hours at places of convivial resort than in the lecture rooms, and extend the Christmas holidays to several weeks, and yet receive credit for attendance on a full course of lectures.

If more than thirty years ago our profession, in a section of country not large, could purchase a lot and erect a commodious structure with all the requirements for thorough instruction in dental science, could not the profession of the country now, with its greatly increased ability and interest in education, establish one or two schools with most ample accommodations and appliances for thorough instruction in dental theory and practice of as many students as are at present in attendance at all our dental institutions?

Thus an able corps of the best teachers could be organized and well rewarded for devoting their entire time and attention to their duties as instructors. The terms could be made to embrace all the year excepting proper (but not long) vacations.

I would prefer such school or schools established at points convenient of access, on their own basis, and not attached to a medical school or university. Dow 3D.

